# Supplementary material for: Holographic Traction Force Microscopy
Source: Sci Rep. 2018 Feb 14;8:3038. doi: 10.1038/s41598-018-21206-2 (PMC5813032; doi:10.1038/s41598-018-21206-2)
Supplement: Supplementary file 1 — Supplementary information [file 41598_2018_21206_MOESM1_ESM.pdf]

Supplementary Information

Holographic Traction Force Microscopy

Stanislaw Makarchuk, Nicolas Beyer, Christian Gaiddon, Wilfried Grange, Pascal Hébraud

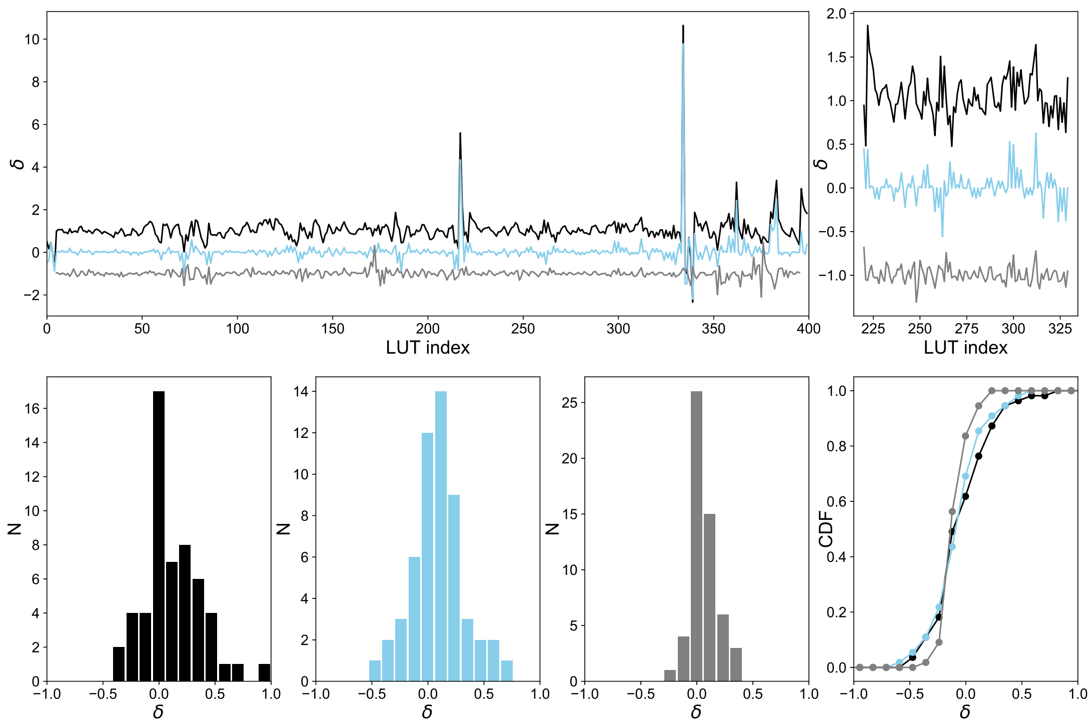

Figure 1. Tracking accuracy along the z direction. Bead images (20 at 20 Hz and then averaged) were acquired for 400 different planes (along z, every 50 nm. Similar to Fig 1 in the main article). A LUT (200 elements) was built using a subset of averaged images (those acquired at position $2i$ only ($[0;199]$)). Then, all averaged images (400) were tracked. Upper panel, left: the difference δ (between the expected and the computed values) in units of LUT indexes computed from square differences (SD, back), square differences and biased corrected (BC, blue [1]) and phase values (PV, grey [3]). For the ease of reading, an offset of +1 (-1) has been added to the SD (PV) values. Upper panel, right: zoom in the region where tracking is best (below the focus). As already shown [1], BC successfully recovers positions for images belonging to the LUT (δ below 1E-3 for every second values). This correction reduces tracking noise but fails to recover positions for images acquired at planes $2i+1$. Lower panel. Shown are histograms for δ values measured at planes $2i+1$. Also shown is the Cumulative Probability Distribution (right panel). This analysis shows that BC only sightly increase tracking accuracy (as compared to SD). This finding should be attributed that the bias [1] does not show a linear dependence over the overall LUT step size. In contrast, PV shows a significant improvement or both SD and BC.

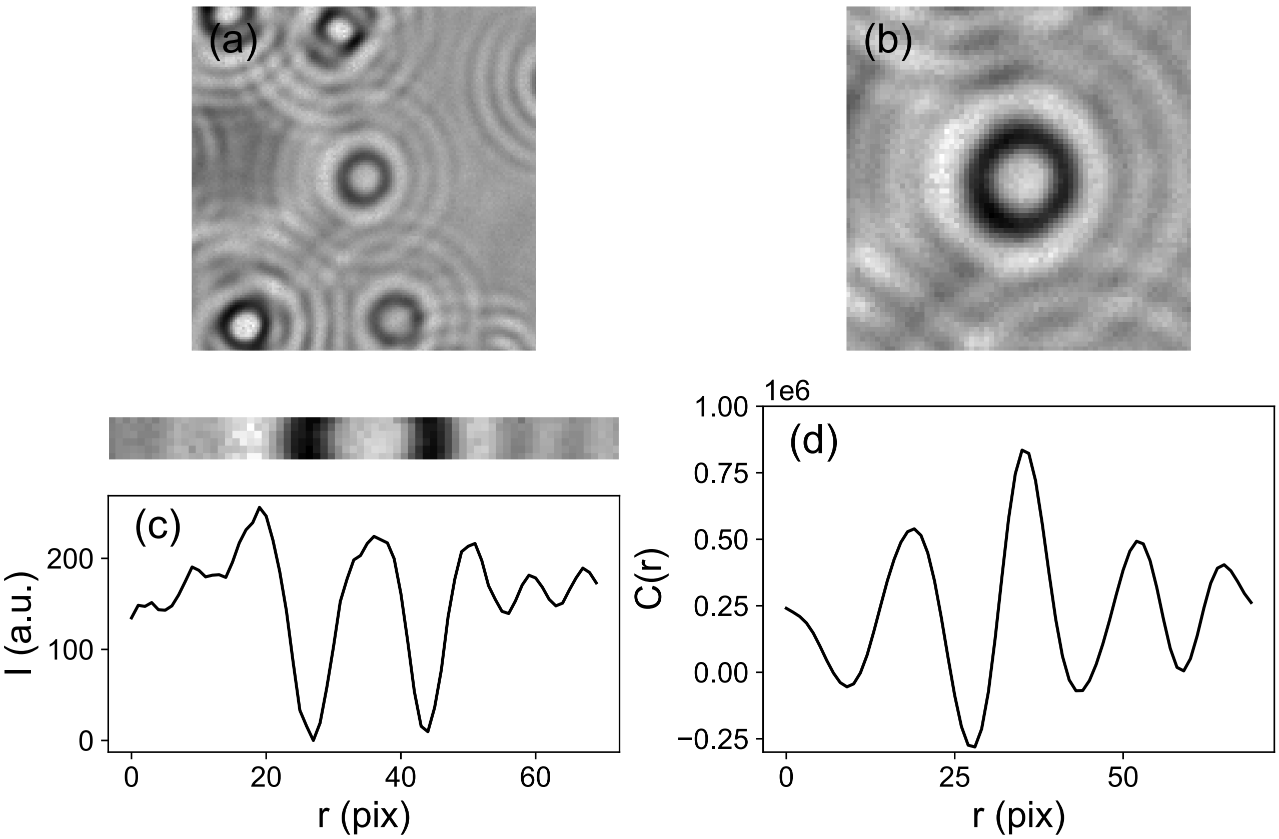

Figure 2. In-plane detection of particles using tracking algorithms of Magnetic Tweezers experiments [2]. **(a)** Same image as in Fig. 1 of the main text, showing a particular region ($141X141$ pixels). **(b)** The approximate positions of the particles are initially manually identified for the first image. For subsequent images, the position is taken from the particles centers at previous time. Then a specific region of interest around the center particle is selected (SxS pixels, S is typically 70 pixels). **(c)** From there, a slice (SxW pixels, W is typically 6 pixels) can be extracted for both directions (shown here is the slice used to determine the x position of the particle). Averaging along W allows to determine a 1-dimensional profile f (bottom). **(d)** The cross correlation ($C(r)$, main text [4]) is calculated from $\mathscr{F}^{-1}(\mathscr{F}^2(\nu))$, where $\mathscr{F}$ denotes the 1-dimensional Fourier Transform of f. Note that $\mathscr{F}^{-1}(\mathscr{F}^2(\nu))$ is equivalent to calculate the cross correlation bewteen $f(x)$ and $f(-x)$ (where the elements of the 1d array are reversed). Fitting the maximum of $C(r)$ with a polynomial function allows to determine the position with sub-pixel accuracy (see Fig. 1 of the main text).

References

1. van Loenhout, M. T., Kerssemakers, J. W., De Vlaminck, I. & Dekker, C. Non-bias-limited tracking of spherical particles, enabling nanometer resolution at low magnification *Biophysical Journal* **102**, 2362–2371 (**2012**).

2. Gosse, C. *et al.* Magnetic Tweezers: Micromanipulation and Force Measurement at the Molecular Level, *Biophysical Journal* **82**, 3314–3329 (**2002**).

3. Lionnet, T. *et al.* Single-molecule studies using magnetic traps, in *Single Molecule Techniques: a Laboratory Manual*, *Cold Spring Harbor Laboratory Press* (**2008**).

4. Heinrich, V *et al.* Imaging Biomolecular Interactions by Fast Three-Dimensional Tracking of Laser-Confined Carrier Particles, in *Single Molecule Techniques: a Laboratory Manual*, *Langmuir* **24**, 1194–1203 (**2008**).
